# Gene Expression Modulation of Markers Involved in Bone Formation and Resorption by Bisphenol A, Bisphenol F, Bisphenol S, and Bisphenol AF

**DOI:** 10.3390/genes15111453

**Published:** 2024-11-11

**Authors:** Enrique García-Recio, Anabel González-Acedo, Francisco Javier Manzano-Moreno, Elvira De Luna-Bertos, Concepción Ruiz

**Affiliations:** 1 Biomedical Group (BIO277), Department of Nursing, Faculty of Health Sciences, University of Granada, Avda. Ilustración 60, 18016 Granada, Spain; egr@ugr.es (E.G.-R.); anabelglez@ugr.es (A.G.-A.); fjmanza@ugr.es (F.J.M.-M.); crr@ugr.es (C.R.); 2 Institute of Biosanitary Research, ibs.Granada, Avda. de Madrid 15, Pabellón de Consultas Externas, 2ª Planta, 18012 Granada, Spain; 3 Biomedical Group (BIO277), Department of Stomatology, School of Dentistry, University of Granada, 18016 Granada, Spain; 4 Institute of Neuroscience, University of Granada, 18016 Granada, Spain

**Keywords:** Bisphenol A, Bisphenol AF, Bisphenol F, Bisphenol S, bone remodeling, gene expression

## Abstract

Background: Bisphenol A (BPA) and its analogs (BPF, BPS, and BPAF) are recognized for inducing detrimental effects on various tissues, including bone. Objectives: The aim of this study is to investigate their impact on information and repair processes, specifically focusing on vascular endothelial growth factor (VEGF), transforming growth factor β1 (TGF-β1), and the receptors for transforming growth factor β (TGFR1, TGFR2, and TGFR3). Methods: Human osteoblasts isolated through primary culture from bone samples of healthy volunteers were subjected to cultivation in the presence of various dosage levels (10^−5^, 10^−6^, or 10^−7^ M) of BPA, BPF, BPS, or BPAF for 24 h. Gene expressions of RANKL, OPG, TGF-β1, TGFR1, TGFR2, TGFR3, and VEGF were analyzed by real-time polymerase chain reaction (RT-PCR). All experiments included untreated cells as controls. Results: Expressions of RANKL and OPG were dose-dependently downregulated by the presence of all tested bisphenols (BPs) except for BPAF, whose presence upregulated OPG expression at all three doses. TGF-β1 expression was downregulated by all BP treatments, and TGF-β1 receptor expression was also downregulated as a function of the BP and dose. VEGF expression was downregulated in the presence of BPF and BPAF at all three doses and in the presence of BPA at the two higher doses (10^−5^, and 10^−6^ M), but it was not changed by the presence of BPS at any dose. Conclusions: The inhibition of both RANKL and OPG by the BPs, with a higher %inhibition of RANKL than of OPG, appears to rule out BP-induced activation of osteoclastogenesis via RANKL/RANK/OPG. Nevertheless, the effect of the BPs on the expression by osteoblasts of TGF-β1, TGF-β receptors, and VEGF indicates that these compounds can be responsible for major molecular changes in this cell population, contributing to their adverse effects on bone tissue.

## 1. Introduction

Bone tissue, as a highly dynamic mineralized connective tissue, reacts consistently to various endogenous stimuli, such as hormones and growth factors, as well as exogenous influences, including environmental and nutritional factors. The process of bone remodeling is intricate, involving a delicate balance between the formation of new bone by osteoblasts and the resorption, or destruction, of bone by osteoclasts. This delicate equilibrium is sustained through an ongoing dialogue between these two types of cells. Numerous molecules contribute to this communication, allowing for the synchronization needed for effective bone remodeling. Among the most critical systems in this process is the osteoprotegerin/receptor activator of the nuclear factor-κ B ligand/receptor activator of the nuclear factor-κ B (OPG/RANKL/RANK) system, which plays a key role in regulating bone homeostasis and remodeling mechanisms through interactions between osteoblasts and osteoclasts [1,2]. Furthermore, osteoblasts are also influenced by other growth factors (GFs), particularly transforming growth factor β (TGF-β1) and vascular endothelial growth factor (VEGF), which modulate various aspects of bone growth and repair [3,4].

Bisphenols are chemical compounds widely used in the production of synthetic polymers, particularly plastics and epoxy resins. Although all bisphenols share a bisphenolic configuration based on two phenol rings, each variant displays structural differences in functional groups, impacting both chemical stability and bioactivity. BPA, historically the most commonly employed and extensively studied bisphenol, is primarily used in the manufacturing of polycarbonate plastics and epoxy resin coatings, leading to its pervasive environmental release through consumer products such as food containers and water bottles [5,6]. BPA is well-known for its endocrine-disrupting capabilities, with multiple studies documenting its adverse health effects across various organisms, which has motivated a ban on its use in consumer products and the search for BPA-free alternatives [7].

In response to these concerns, BPF and BPS were introduced as potential substitutes for BPA in consumer applications, appearing frequently in products labeled “BPA-free”. However, their structural and functional similarities suggest comparable toxicological risks, as their endocrine activity has been reported in several studies [8]. BPS, for instance, exhibits higher hydrolytic stability than BPA, potentially leading to prolonged environmental persistence, particularly in aquatic systems [9]. BPF, structurally analogous to BPA, also demonstrates reduced degradability, thus increasing its potential for bioaccumulation in organisms and sediments [10]. BPAF, in contrast, is less common in consumer products but has industrial significance due to its incorporation of two trifluoromethyl groups, which impart rigidity and may enhance endocrine-disrupting potency relative to other bisphenols [9]. This structural variation is associated with potentially increased affinity for hormonal receptors, making BPAF a compound of particular interest in toxicity studies [11].

The environmental sources of these bisphenols include industrial effluents, leaching from plastic materials in landfills, and the degradation of consumer goods in contact with food, among others [7]. Due to their variable degradation resistance, these compounds tend to adsorb to soil particles and aquatic sediments, facilitating bioaccumulation in food webs and ultimately increasing indirect human exposure [12]. This differentiated environmental behavior underscores the necessity of analyzing their relative persistence and toxicity to understand their potential impact on health and ecosystems.

Environmental contaminants like BPA and its analogs can interfere with bone homeostasis through a variety of mechanisms. These include hormonal imbalances, direct cytotoxic effects on osteoblasts, and changes in osteoclast activity, all of which can promote the development of bone diseases such as osteopenia or osteoporosis [13,14,15]. The relationship between BPA exposure and sex hormones is particularly complex and may carry significant biological and clinical implications for bone, which is a target organ for sex hormones. Although BPA has traditionally been considered a weak xenoestrogen due to its lower binding affinity to estrogen nuclear receptors compared to 17-β estradiol, recent studies have shed light on its nuanced interactions with estrogen receptors (ERs). Estrogen receptors are divided into two subtypes, ER-α and ER-β. ER-α is predominantly expressed in the endometrium, breast cancer cells, ovarian stroma, and hypothalamus, whereas ER-β is found in the kidney, brain, lung, heart, intestine, prostate, endothelial cells, and importantly, bone tissue [16]. BPA has been shown to bind to both ER-α and ER-β, with a higher affinity for ER-β, although its binding to these receptors is distinct from that of estradiol, potentially resulting in different intracellular effects. In certain cell types, BPA demonstrates agonistic activity via ER-β, while exhibiting a mixed profile of agonistic and antagonistic activity via ER-α [16,17,18]. Additionally, a newly identified nuclear receptor, ER-γ, has been shown to bind to BPA with high affinity, although its precise function and natural ligand remain unknown [19,20]. Studies suggest that BPA may trigger estrogen-like effects at low doses through non-classical pathways, possibly involving ER-γ [19,21]. Moreover, BPA has been found to induce inflammatory responses by stimulating the production of proinflammatory cytokines and inhibiting anti-inflammatory cytokine production. It can also elevate reactive oxygen species (ROS) levels, leading to oxidative DNA damage and cell death, which in turn may further exacerbate inflammation [14,22]. The combination of inflammation and oxidative stress caused by BPA exposure is thus hypothesized to negatively impact bone health. Several authors have reported that both BPA and its analogs exert deleterious effects on the two primary cell types involved in bone remodeling, osteoblasts, and osteoclasts, through mechanisms involving a reduction in bone morphogenetic protein-2 (BMP-2) and alkaline phosphatase (ALP) activity, as well as the disruption of bone metabolism via the RANKL, apoptosis, and Wnt/β-catenin signaling pathways [23,24]. 

Studies have shown that the culture of human osteoblasts in the presence of BPA, BPF, BPS, or BPAF leads to significant alterations in their growth, osteogenic gene expression, ALP synthesis, and mineralization [25,26,27]. 

Given the involvement of RANKL, OPG, and GFs (e.g., TGF-β1 or VEGF) in bone remodeling, consisting of the removal of mineralized bone by osteoclasts, previously activated by osteoblasts through RANKL and regulated by OPG synthesized by osteoblasts, followed by the formation of bone matrix by osteoblasts, which is subsequently mineralized, involving various growth factors such as TGF and VEGF, so the aim of this study was to evaluate the effect of BPA and three of its analogs (BPF, BPS, and BPAF) on the gene expression of RANL, OPG, TGF-β1, and its receptors (TGFR1, TGFR2, TGFR3) and VEGF involved in the bone remodeling process, by cultured human osteoblasts.

## 2. Materials and Methods

### 2.1. Chemicals

All bisphenols used in this study, including BPA, BPF, BPS, and BPAF, were provided by Sigma-Aldrich (St. Louis, MO, USA) In order to use them, they had to be dissolved in a concentration ≤ 0.05% dimethyl sulfoxide (DMSO) for all experiments. 

### 2.2. Isolation and Primary Culture of Human Osteoblasts

The isolation and establishment of human osteoblast lines followed the protocols previously described by García-Martínez et al. (2011) and Manzano-Moreno et al. (2013) [28,29]. Human bone tissue samples were obtained from healthy participants who attended the Clinic of the Faculty of Dentistry of the University of Granada (Spain) to undergo mandibular osteotomy or extraction of lower wisdom teeth. To remove periosteal debris and bone marrow from the fragments, a phosphate-buffered saline solution was used to wash them thoroughly. The bone fragments were then seeded into culture flasks and cultured according to the protocols mentioned above. Ultimately, three primary osteoblast cell lines were established from each source. Each cell line was cultured independently for subsequent experiments. All participants gave their written informed consent to participate in the research, which had the approval of the university’s research ethics committee (reg. No. 524/CEIH/2018, approved date 16 April 2018).

### 2.3. Treatments

Human osteoblast cell lines were exposed to BPA, BPF, BPS, or BPAF at concentrations of 10^−5^, 10^−6^, or 10^−7^ M for 24 h. Untreated osteoblasts served as the control group for comparison.

### 2.4. Effect of BPA, BPF, BPS, and BPAF on RANKL, OPG, TGF-β1, TGFR1, TGFR2, TGFR3 and VEGF Gene Expression of Human Osteoblasts

Real-time polymerase chain reaction (RT-PCR) was employed to assess the impact of bisphenols (BPs) on the gene expression of RANKL, OPG, TGF-β1, and its receptors (TGFR1, TGFR2, TGFR3) and VEGF in cultured human osteoblasts. Following 24 h of osteoblast culturing with each BP at the respective doses, cells were detached from the culture flask using 0.05% trypsin-EDTA solution (Sigma -Aldrich; St. Louis, MO, USA) and individually harvested. Messenger RNA (mRNA) was then extracted using the Qiagen RNeasy extraction kit (Qiagen, Inc.), a silicate gel technique including a DNAase digestion step. The mRNA amount was measured by UV spectrophotometry at 260 nm (Eppendorf AG). Subsequently, an equal amount of RNA (1 μg of mRNA from each group was brought to 40 μL of total volume) was reverse-transcribed to complementary DNA (cDNA) and amplified using the iScript™ cDNA Synthesis Kit (Bio-Rad Laboratories) by means of the polymerase chain reaction according to the manufacturer’s instructions (or in accordance with the manufacturer’s instructions). Primers were designed using the NCBI-Nucleotide library and its Primer-BLAST tool (Table 1); all primers were matched to the mRNA sequences of the target genes (NCBI Blast software 2.2.25). 

The real-time quantitative polymerase chain reaction (RT-qPCR) was performed with the SsoFastTM EvaGreen^®^ Supermix Kit (Bio-Rad laboratories), following a previously outlined protocol [26]. The comparative Ct method was employed for the relative quantification of gene expression. mRNA levels for each gene were measured in ng of mRNA per average ng of housekeeping mRNAs [30]. cDNA from each individual cell experiment was analyzed in triplicate using RT-PCR. The results were presented as a percentage of expression relative to the control group.

### 2.5. Statistical Analysis

The SPSS 22.0 statistical package (IBM, Armonk, NY, USA) was used as a tool for statistical analysis of the data. Results were reported as mean ± standard deviation (SD). The normality of the distribution of variables was tested using the Kolmogorov–Smirnov test, and ANOVA with Bonferroni correction was applied for multiple comparisons. For all tests, a value of *p* < 0.05 was set as the level of statistical significance. Experiments were performed with three different primary human osteoblast cell lines, and assays were performed in triplicate.

## 3. Results

### 3.1. Effect of BPs on the Modulation of RANKL and OPG Gene Expression 

**Figure 1:**
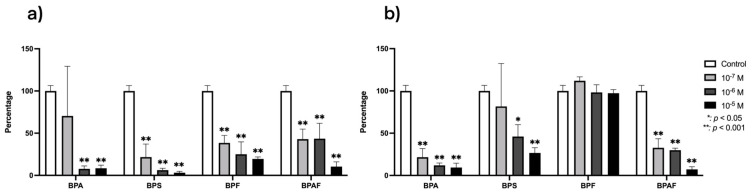
Expression of RANKL (**a**) and OPG (**b**) in primary human osteoblasts treated for 24 h with BPA, BPS, BPF, and BPAF (10^−7^, 10^−6^, and 10^−5^ M). Data are expressed as a percentage relative to the expression levels in control cells. Significant differences: * *p* ≤ 0.05; ** *p* ≤ 0.001.

The results regarding the effects of BPA and its analogs on the gene expression of RANKL and OPG are depicted in Figure 1. The expression of RANKL expression was downregulated (*p* ≤ 0.0001) by treatment with all BPs except for BPA at the lowest dose (10^−7^ M), which had no significant effect. OPG expression was also downregulated by the presence of BPA or BPAF at all tested doses and by BPS at the highest doses (10^−5^ and 10^−6^ M), and it was upregulated by the presence of BPF. The percentage downregulation of expression in the presence of BPA and BPS was higher for RANKL than for OPG and vice versa in BPAF (Figure 1).

### 3.2. Effect of BPs on the Modulation of TGF-β1 and Its Receptors Gene Expression

**Figure 2:**
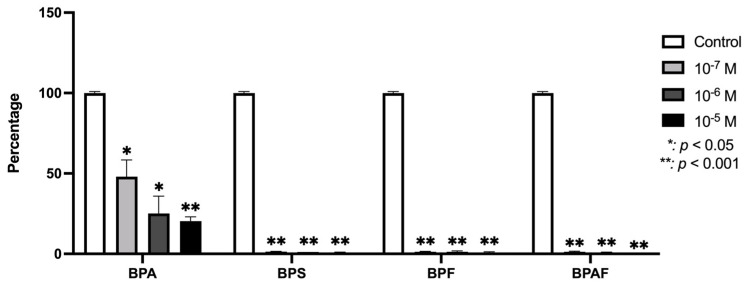
Expression of TGF-β1 in primary human osteoblasts treated for 24 h with BPA, BPS, BPF, and BPAF (10^−7^, 10^−6^, and 10^−5^ M). Data are expressed as a percentage relative to the expression levels in control cells. Significant differences: * *p* ≤ 0.05; ** *p* ≤ 0.001.

**Figure 3:**
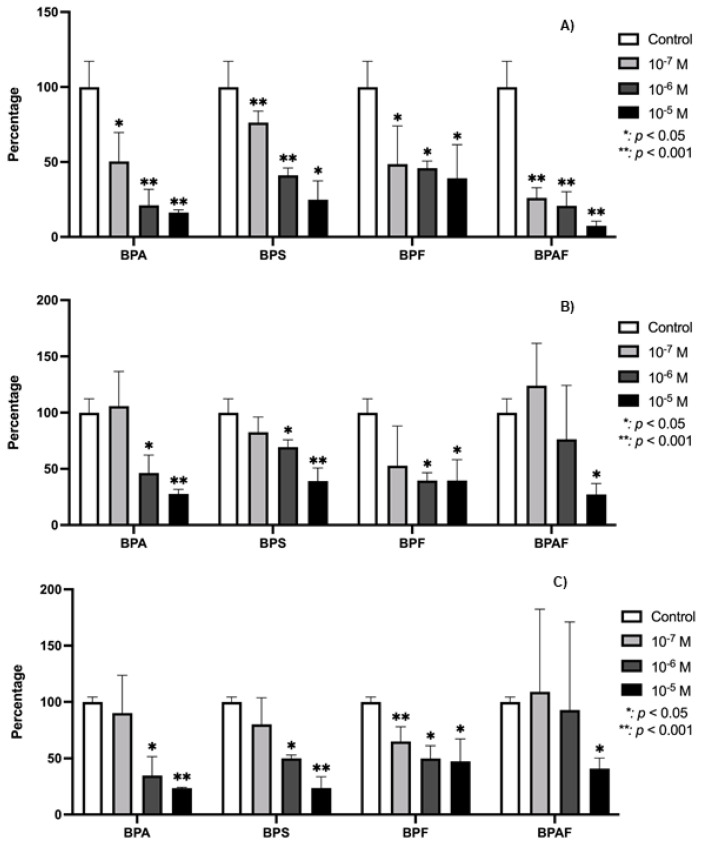
Expression of TGFB-R1 (**A**), TGFB-R2 (**B**), and TGFB-R3 (**C**) in primary human osteoblasts treated for 24 h with BPA, BPS, BPF, and BPAF (10^−7^, 10^−6^, and 10^−5^ M). Data are expressed as a percentage relative to the expression levels in control cells. Significant differences: * *p* ≤ 0.05; ** *p* ≤ 0.001.

The expression of TGF-β1 in human osteoblasts was significantly (*p* ≤ 0.0001 and *p* ≤ 0.05) downregulated by the presence of each BP studied (Figure 2). Figure 3 depicts changes in the expression of TGF-β1 receptors in the presence of BPA, BPF, BPS, or BPAF. TGFR1 expression was downregulated in the presence of all four BPs. TGFR2 was significantly inhibited at the two higher doses of BPA, BPS, and BPF, while their expression was only significantly downregulated by BPAF at the highest dose. Regarding TGFR3, it was significantly inhibited by BPF at all doses, while its expression was significantly downregulated by BPA and BPS only at the highest dose. BPAF significantly altered the expression only at the highest dose.

### 3.3. Effect of BPs on the Modulation of VEGF Gene Expression

**Figure 4:**
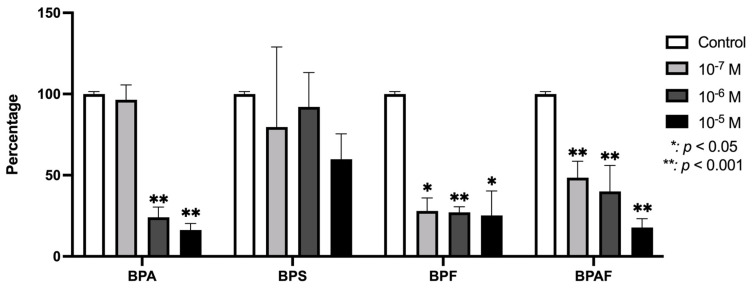
Expression of VEGF in primary human osteoblasts treated for 24 h with BPA, BPS, BPF, and BPAF (10^−7^, 10^−6^, and 10^−5^ M). Data are expressed as a percentage relative to the expression levels in control cells. Significant differences: * *p* ≤ 0.05; ** *p* ≤ 0.001.

Figure 4 illustrates the VEGF expression levels in osteoblasts cultured under the influence of BPA and its analogs. As shown, both BPF and BPAF caused a significant reduction in VEGF expression across the tested conditions. In contrast, BPA demonstrated a significant inhibition of VEGF expression, but only at the higher concentrations used in the study. BPS did not produce any statistically significant changes in VEGF expression at any of the doses tested.

## 4. Discussion

Humans are continuously and ubiquitously exposed to bisphenols (BPs), not only due to their widespread presence in food containers and utensils but also through various other products in our environment. This persistent exposure is a consequence of the extensive use of BPs in industrial applications, where their chemical properties are leveraged to produce plastics, resins, and coatings. Over time, these materials leach bisphenols into food, water, and even the air, resulting in chronic exposure to low doses of these compounds. BPs are of particular concern due to their endocrine-disrupting properties, which can lead to adverse effects across various tissues in the body, including bone. Previous research has indicated that BPs can interfere with bone metabolism by altering the physiology of both osteoblasts and osteoclasts, two critical cell types responsible for maintaining bone homeostasis. Disruption in the balance of bone formation and resorption can lead to bone fragility, highlighting the importance of understanding the molecular pathways affected by BPs. 

The pharmacokinetics of bisphenols may vary depending on the specific compound and the biological system involved but generally follow a similar pattern characterized by rapid absorption and distribution, with limited bioaccumulation due to efficient hepatic metabolism and renal excretion [31,32]. Bisphenols are primarily metabolized in the liver via glucuronidation and sulfation pathways, which produce more hydrophilic metabolites that are excreted in urine and bile. However, certain tissues may experience prolonged exposure, particularly in cases of repeated or high-dose exposure, as bisphenols and their metabolites have demonstrated the ability to bind to proteins and other cellular components, potentially allowing for localized effects [33]. The exposure of osteoblasts to bisphenols in vivo would likely be influenced by systemic levels achieved through environmental or dietary sources. Blood and tissue levels of bisphenols, such as BPA, have been documented in the range of nanomolar to low micromolar concentrations, particularly in individuals with high levels of exposure, such as industrial workers or individuals frequently exposed to plastic products [34]. In this context, the concentrations used in this study (10^−5^, 10^−6^, and 10^−7^ M) are within the upper range of possible exposure levels, aligning with doses shown in previous studies to elicit cellular responses in endocrine-sensitive cells, including osteoblasts [10,11,35,36].

Clinically, the observed effects of bisphenols on osteoblast activity could have implications for bone health, particularly in populations with chronic exposure to bisphenols. Given that bisphenols act as endocrine disruptors, their interference with osteoblast function may contribute to altered bone homeostasis and remodeling, especially in susceptible individuals or those with pre-existing conditions affecting bone metabolism. While further in vivo studies are necessary to confirm these findings, the potential for bisphenols to impact bone integrity suggests that these effects could indeed be clinically significant, particularly over long-term exposure [37,38].

The present in vitro study aimed to explore the impact of BPA and its commonly used analogs (BPF, BPS, and BPAF) on key markers of bone remodeling, focusing on the gene expression of RANKL, OPG, TGF-β1, its receptors (TGFR1, TGFR2, and TGFR3), and VEGF in human osteoblasts. Bone tissue is a complex structure that undergoes continuous formation and resorption to maintain its integrity. This process involves two primary cell populations, osteoblasts, and osteoclasts, with RANK/RANKL/OPG molecules playing a regulatory role in signaling for bone resorption, while TGFβ1 contributes to promoting osteoblast proliferation and differentiation [39,40,41]. Therefore, modulation of any of these molecules may have significant effects on this process and potentially impact bone health. Our findings offer new insights into the disruptive potential of these chemicals on bone homeostasis, highlighting the importance of understanding their long-term effects on human health. They suggest a negative effect on the regulation of osteoclastogenesis, potentially leading to a decreased ability of the bone to regulate osteoclast activity through OPG. Downregulation of these key molecules could lead to a disruption of the normal coupling between bone resorption and formation, contributing to pathological conditions such as osteoporosis.

RANKL, an osteoblastic molecule essential for the activation of osteoclasts, facilitates the differentiation and maturation of these bone-resorbing cells by binding to its receptor (RANK) on the surface of pre-osteoclasts. This interaction promotes osteoclastogenesis, ultimately leading to increased bone resorption and degradation of bone tissue. In contrast, OPG functions as a decoy receptor, produced by osteoblasts through the activation of the Wnt/β-catenin signaling pathway, which binds to RANKL and prevents it from interacting with RANK. This effectively inhibits osteoclastogenesis and helps to maintain the equilibrium between bone formation and resorption [2,41,42]. The significant downregulation of RANKL observed in the current study suggests a potential suppression of osteoclastogenesis, which could inhibit bone resorption. However, the downregulation of OPG, although less pronounced, complicates this interpretation, as a reduction in OPG levels could still allow for unchecked osteoclast activation if sufficient RANKL is present. Notably, BPF was the only bisphenol that did not significantly downregulate OPG expression, suggesting a potentially differential impact of bisphenol analogs on bone metabolism. Given that the RANKL/OPG ratio is a critical determinant of bone resorption, the observed alterations may not necessarily lead to increased osteoclast activity, but they do highlight the complex regulatory role these molecules play in maintaining bone integrity [2,42]. 

RANKL and OPG expression is regulated by a multitude of factors, including hormonal signals (both steroid and peptide hormones), growth factors (GFs), proinflammatory cytokines, and specific transcription factors such as cbfa-1 [43,44,45]. It has been well-established that bisphenol exposure can interfere with these regulatory factors, leading to altered expression of key bone-related genes [46]. In addition, both in vivo and in vitro studies have consistently shown that BPA and its analogs can act as prooxidants, leading to the production of reactive oxygen species (ROS) and the induction of oxidative stress [22,47,48,49]. It is established that oxidative stress at the bone level increases RANKL expression and decreases OPG expression, thereby promoting osteoclastogenesis and bone resorption. Romagnoli et al. (2013) [50] further supported this by demonstrating that oxidative stress enhances RANKL upregulation and OPG downregulation via the activation of protein kinases such as ERK1/2 and JNK. However, in the present study, BPs were found to reduce both RANKL and OPG expression, suggesting that they do not act through this oxidative stress-mediated pathway in promoting osteoclastogenesis.

Exposure to bisphenols also altered the expression of TGF-β1 and its receptors, which play a crucial role in bone remodeling. TGF-β1 is known to stimulate the recruitment of osteoblasts [2,51]. It acts as a key signal in regulating the proliferation, differentiation, and bone formation of osteoblasts [52,53]. The signaling pathway of this growth factor (GF) operates through a surface receptor complex composed of transmembrane glycoproteins with serine–threonine kinase domains on the intracellular side. In the absence of a ligand, the receptors remain as monomers, but when TGFβ1 binds to TGFB-R2, which is phosphorylated in its basal state, it recruits TGFB-R1, forming a complex with both receptors and the ligand. TGFB-R2 then phosphorylates TGFB-R1 on serine and threonine residues, initiating the intracellular signaling cascade. TGFB-R3, in turn, enhances the affinity of TGFB R2 for the ligands [54,55,56]. Given the coordinated action of these receptors, changes in the expression of individual receptors may affect the bone formation and repair phase.

The decrease observed in the expression of TGF-β1 in the presence of the BPs studied is related to previously reported data, where it is described how these substances inhibit proliferation, differentiation, and mineralization. Similarly, they inhibit the gene expression of different osteogenic markers [24,25,26,27,57]. 

All tested BPs were observed to downregulate the expression of VEGF, a key growth factor involved in the regulation of vascular development and angiogenesis, with the exception of BPS at the doses studied. VEGF plays a crucial role in the maintenance and repair of bone, which is a highly vascularized tissue. Specifically, VEGF is essential for promoting angiogenesis, facilitating both skeletal development and bone healing 4. Osteoblasts, which are responsible for bone formation, serve as one of the primary sources of VEGF in bone tissue, where this growth factor plays multiple roles during different stages of the bone repair process. By stimulating the formation of new blood vessels, VEGF ensures the delivery of oxygen and essential nutrients to bone cells, supporting their growth and function. In addition to its angiogenic effects, VEGF also promotes the differentiation of osteoblasts, guiding their maturation into fully functional cells capable of synthesizing and mineralizing the bone matrix. In this way, VEGF stimulates angiogenesis, osteoblast differentiation, and the synthesis of molecules involved in bone remodeling (RANKL/OPG) and formation (TGF-β1 and VEGF) at the repair site, while it inhibits chondrogenesis and the proliferation of osteoblast progenitors [4,58,59]. The ability of VEGF to modulate these processes underscores its importance not only in angiogenesis but also in maintaining overall bone health. The present in vitro findings, which demonstrate a downregulation of VEGF in response to BP exposure, may partially explain the observed decrease in RANKL and OPG expression, as well as the inhibition of osteoblast proliferation. This suggests that BPs may exert both direct and indirect adverse effects on bone tissue, particularly through their interference with VEGF-mediated pathways.

Furthermore, endocrine, inflammatory, and oxidative factors have been implicated in the effects of BPs on bone tissue [14]. This study supports previous findings that BPs directly impact osteoblast physiology, impairing their functional capacity, in line with earlier research [25,26,27]. The destructive influence of BPs on bone cells, coupled with their potential to induce oxidative stress, underscores their significant role in bone degradation.

The limitation of the study is that we worked with cell cultures under defined conditions, which may not be directly extrapolated to an in vivo situation without further validation. Although in vitro studies provide valuable insights, the complexities of a living organism, where multiple physiological factors interact, require additional confirmation.

The in vitro results from this study highlight the potential of bisphenols to significantly alter the gene expression of critical molecules involved in bone remodeling, specifically RANKL, OPG, TGF-β1, its receptors, and VEGF, in primary human osteoblasts. In an in vivo setting, these molecular changes could translate into disruptions in bone homeostasis, given the central role of these markers in regulating osteoblast and osteoclast activity. For instance, the downregulation of RANKL by bisphenols observed in this study may inhibit osteoclast differentiation and function, potentially reducing bone resorption. However, the concurrent downregulation of OPG, particularly with BPA and BPS, complicates this effect by potentially allowing for some degree of osteoclast activation, even in the presence of lower RANKL levels. This dual downregulation effect could disrupt the delicate RANKL/OPG balance that is essential for bone turnover, potentially leading to weakened bone structure if such changes are sustained in vivo. The downregulation of TGF-β1 and its receptors, as well as VEGF, suggests additional avenues by which bisphenols could impair bone formation and repair in a living organism. TGF-β1 plays a critical role in osteoblast proliferation, differentiation, and matrix synthesis, which are necessary for effective bone remodeling and regeneration. In an in vivo context, reduced TGF-β1 signaling could weaken osteoblast functionality, thereby impacting bone density and structural integrity. Similarly, VEGF is crucial for vascularization within bone tissue, which is essential for delivering nutrients and removing waste products. Impaired VEGF expression, as observed in response to BPA, BPF, and BPAF, could potentially lead to reduced bone perfusion, delaying or impairing the healing process and compromising bone health, particularly in scenarios of repeated or chronic exposure to bisphenols.

This study aims to reinforce existing findings on the effects of BPS on osteoblast growth, differentiation, and mineralization. However, it would also be beneficial to evaluate the impact on osteoclasts and to examine the in vivo effects of BPs on bone tissue, as both cell populations are crucial for maintaining bone homeostasis.

Although this study focused exclusively on the effects of BPA and its analogs (BPF, BPAF, and BPS) on the gene expression of RANKL, OPG, VEGF, TGFβ1, and TGFβ1 receptors, future studies should investigate how these effects translate directly to the synthesis of the molecules under investigation.

Future research should deepen the understanding of the interactions of bisphenols with different cell populations in co-culture systems, such as osteoclasts, osteoprogenitors, and even endothelial cells. Studying these complex cellular interactions and the molecular pathways involved, including those related to oxidative stress, will provide a more comprehensive view of the impact of BPs on bone health. In addition, identifying safer BP alternatives with fewer endocrine-disrupting properties could help reduce human exposure and associated health risks.

## 5. Conclusions

The findings of this study demonstrate that bisphenols, including BPA, BPF, BPS, and BPAF, exert deleterious effects on bone cells by modulating the expression of key genes involved in bone remodeling. Specifically, a reduction in RANKL and OPG suggests a direct disruption of the balance between bone formation and resorption, as RANKL promotes osteoclast formation, leading to bone resorption, while OPG acts as a decoy receptor that inhibits this activity, maintaining bone integrity. Additionally, a decrease in TGF-β1, along with its receptors, indicates a reduction in the anabolic processes necessary for bone formation and repair. The coordinated action of TGF-β1 and its receptors is essential for osteoblast proliferation and differentiation, key steps in the bone formation process. VEGF, which plays an essential role in angiogenesis and nutrient supply to bone tissue, is also downregulated by bisphenols. These findings highlight how dysregulation of these molecules and receptors may destabilize the homeostatic balance of bone tissue, potentially leading to significant long-term consequences for bone health.

## Figures and Tables

**Table 1:** Sequence of target gene primers for the amplification of cDNA by real-time PCR.

Gene	Sense Primer	Antisense Primer
*RANKL*	ATACCCTGATGAAAGGAGGA	GGGGCTCAATCTATATCTCG
*OPG*	ATGCAACACAGCACAACATA	GTTGCCGTTTTATCCTCTCT
*TGF-β1*	TGAACCGGCCTTTCCTGCTTCTCATG	GCGGAAGTCAATGTACAGCTGCCGC
*TGFB-R1*	ACTGGCAGCTGTCATTGCTGGACCAG	CTGAGCCAGAACCTGACGTTGTCATATCA
*TGFB-R2*	GGCTCAACCACCAGGGCATCCAGAT	CTCCCCGAGAGCCTGTCCAGATGCT
*TGFB-R3*	ACCGTGATGGGCATTGCGTTTGCA	GTGCTCTGCGTGCTGCCGATGCTGT
*VEGF*	CCTTGCTGCTCTACCTCCAC	CACACAGGATGGCTTGAAGA

## Data Availability

The data supporting this study are available upon request.
